# Effective use of high CO_2_ efflux at the soil surface in a tropical understory plant

**DOI:** 10.1038/srep08991

**Published:** 2015-03-11

**Authors:** Atsushi Ishida, Takashi Nakano, Minaco Adachi, Kenichi Yoshimura, Noriyuki Osada, Phanumard Ladpala, Sapit Diloksumpun, Ladawan Puangchit, Jin Yoshimura

**Affiliations:** 1 Center for Ecological Research, Kyoto University, Shiga 520-2113, Japan; 2 Mount Fuji Research Institute, Yamanashi 403-0005, Japan; 3 Institute of Industrial Science, The University of Tokyo, Komaba, Tokyo 153-8505, Japan; 4 Kansai Research Center, Forestry and Forest Products Research Institute, Kyoto 612-0855, Japan; 5 Tomakomai Experimental Forest, Field Center for Northern Biosphere, Hokkaido University, Tomakomai 053-0035, Japan; 6 National Park, Wildlife and Plant Conservation Department, Bangkok 10900, Thailand; 7 Faculty of Forestry, Kasetsart University, Bangkok 10900, Thailand; 8 Graduate School of Science and Technology, and Department of Mathematical and Systems Engineering, Shizuoka University, Shizuoka 432-8561, Japan; 9 Department of Environmental Science and Forest Biology, State University of New York College of Environmental Science and Foestry, Syracuse, NY 13210, USA; 10 Marine Biosystems Research Center, Chiba University, Kamogawa, Chiba 299-5502, Japan

## Abstract

Many terrestrial plants are C_3_ plants that evolved in the Mesozoic Era when atmospheric CO_2_ concentrations ([CO_2_]) were high. Given current conditions, C_3_ plants can no longer benefit from high ambient [CO_2_]. *Kaempferia marginata* Carey is a unique understory ginger plant in the tropical dry forests of Thailand. The plant has two large flat leaves that spread on the soil surface. We found a large difference in [CO_2_] between the partly closed space between the soil surface and the leaves (638 µmol mol^−1^) and the atmosphere at 20 cm above ground level (412 µmol mol^−1^). This finding indicates that the plants capture CO_2_ efflux from the soil. Almost all of the stomata are located on the abaxial leaf surface. When ambient air [CO_2_] was experimentally increased from 400 to 600 μmol mol^−1^, net photosynthetic rates increased by 45 to 48% under near light-saturated conditions. No significant increase was observed under low light conditions. These data demonstrate that the unique leaf structure enhances carbon gain by trapping soil CO_2_ efflux at stomatal sites under relatively high light conditions, suggesting that ambient air [CO_2_] can serve as an important selective agent for terrestrial C_3_ plants.

The geological record indicates that the C_3_ land plants originated during the middle to late Ordovician period (450 to 440 million years ago) when atmospheric CO_2_ concentrations ([CO_2_]) were still very high (approximately 4% compared with 0.039% at present) and O_2_ concentrations ([O_2_]) in air were low (approximately 15% compared with 21% at present)^1^,^2^. Although the down-regulation of Rubisco (ribulose-1,5-bisphosphate carboxylase/oxygenase) under high [CO_2_] is a well-known phenomenon^3^, high [CO_2_] and low [O_2_] in the ancient air would have contributed to an increase in carbon assimilation rates (*A*) due to the kinetics of Rubisco. A meta-analysis of FACE (free-air CO_2_ enrichment) experiments revealed that the average maximum carboxylation rates under doubled [CO_2_] were −17% in C_3_ crops and −4% in C_3_ trees due to dawn-regulation. On average, the increase in light-saturated net photosynthesis under doubled [CO_2_] was 13% in C_3_ crops and 47% in C_3_ trees^3^. This finding may indicate that C_3_ plants in the past exhibited increased carbon (C) gain and that more extensive C cycling occurred in forest ecosystems compared with the present era. During the Cenozoic era, atmospheric O_2_ concentrations increased and atmospheric [CO_2_] became largely depleted, with record minimum [CO_2_] during the Oligocene/Miocene epoch (24 million years ago)^4^. Since the advent of the Industrial Revolution, atmospheric [CO_2_] has increased rather rapidly due to the modernization of human society and increasing reliance on coal and oil burning. In the photosynthetic CO_2_-response curves of C_3_ plants, the transition of the limitation from ribulose-1,5-bisphosphate (RuBP) carboxylation limitation to RuBP regeneration limitation is typically observed between ambient and doubled ambient [CO_2_]^5^. Thus, C_3_ plants are constrained by the carboxylation limit of RuBP in the present-day air [CO_2_]. In contrast, photosynthesis in C_4_ plants is not limited by low air [CO_2_]^6^ because these plants possess the appropriate enzyme (PEP carboxylase) and the specific anatomy in bundle sheath cells required to increase the CO_2_ partial pressure around Rubisco sites^7^. C_4_ plants have evolved to improve plant carbon and water relations simultaneously during photosynthesis and to cope with declining atmospheric [CO_2_] and increasing water demand^4^,^8^,^9^. However, C_3_ plants have not evolved carbon-concentrating mechanisms in their physiology and anatomy.

Even in present-day ecosystems, sites with high air [CO_2_], such as forest floors^10^ and volcanic vents^11^ are observed. The high [CO_2_] found on forest floors originates from the respiration of soil organisms and plant-root systems. Attention has been focused on the large contributions of sunflecks or sun patches to net C assimilation rates (*A*) in forest understory plants, indicating strong light limitation^12^,^13^. However, the potential effects of rising [CO_2_] on *A* in understory plants have rarely been evaluated. High [CO_2_] should contribute to the survival of understory plants that experience reduced photosynthetic rates due to water stress^14^. The stable carbon isotope ratios of understory plants indicate that these plants re-fix the efflux C in tropical^15^ and cool-temperate forests^16^.

High [CO_2_] that originates from the soil surface dissipates rapidly due to diffusion and mass flow caused by wind. Although wind velocity is reduced near the understory, an extremely gentle breeze is sufficient to diffuse CO_2_ from the soil surface^17^. Therefore, for understory plants to effectively use this high soil-efflux [CO_2_], they must trap CO_2_ near the soil surface. In the present study, we report the discovery of an understory ginger plant, *Kaempferia marginata* Carey (Zingiberaceae), which effectively traps soil-efflux CO_2_ in the closed space between the soil surface and its leaves. This plant enhances photosynthesis by 45 to 48% under relatively high light conditions. It is a drought-deciduous, perennial herb found in tropical dry forests in Southeast Asia. Based on measurements of ambient air [CO_2_], photosynthetic capacity, and the stable carbon isotope ratios in the lamina, we demonstrate that this ginger plant makes effective use of high [CO_2_] on the forest floor.

## Results

**Figure 1:**
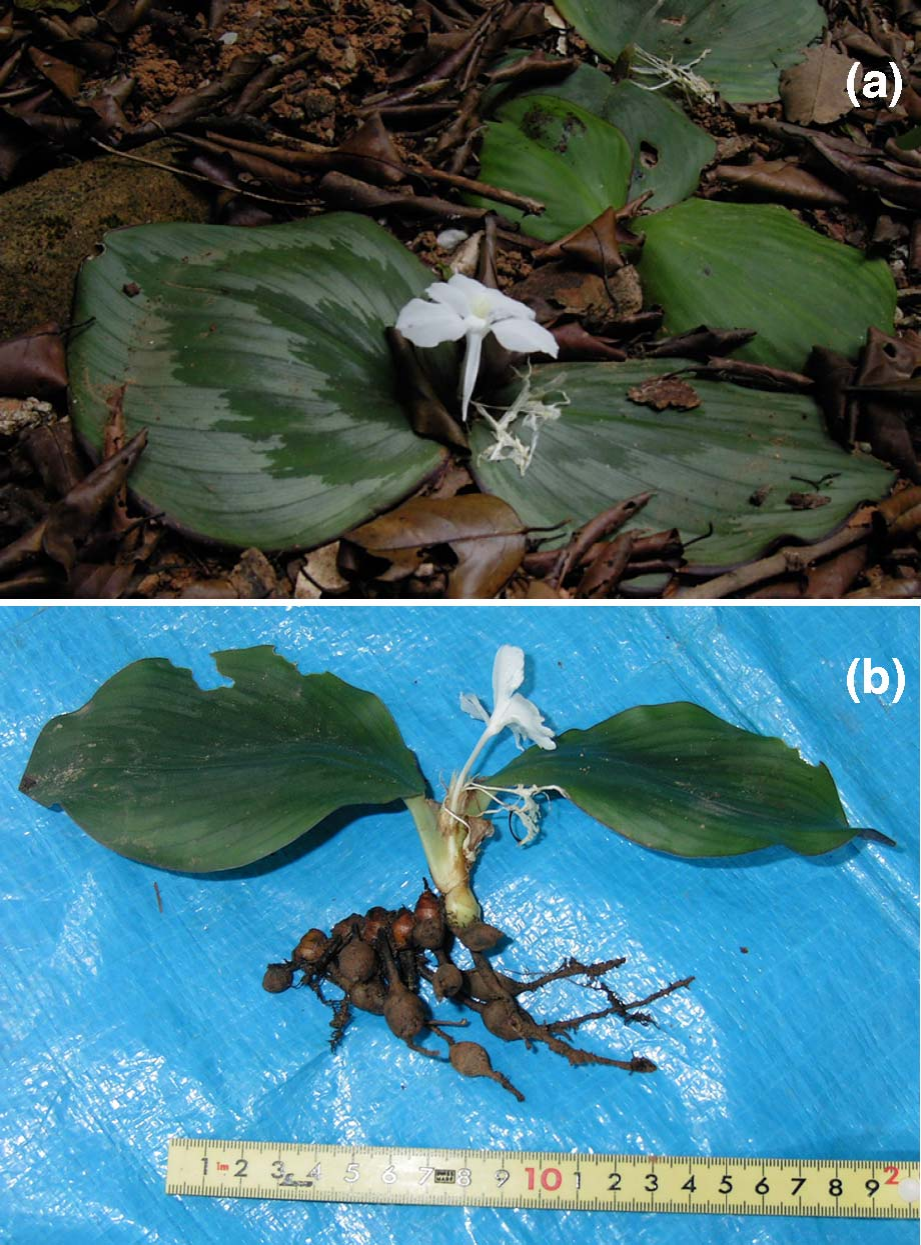
An understory ginger plant, *Kaempferia marginata* Carey, with a unique leaf structure in a tropical forest in Southeast Asia. (a) field-grown plants, (b) A plant removed from the soil; two large leaves and a poor root system are evident.

The ginger plant has a unique leaf structure; the individual plant has two flat leaves that spread on the soil surface, and the leaf edges are often curled downward to capture the air under its leaf blades (Fig. 1). The root system is small, indicating that this plant has a poor water uptake capacity. The uppermost height of a single leaf blade is only 24 mm above the ground surface on average and defines a relatively closed space between the leaf blade and the soil surface (Table S1). The stomatal densities were 1.6 mm^−2^ and 20.9 mm^−2^ on the adaxial and abaxial leaf surfaces, respectively, indicating that approximately all stomata face the soil surface. The distributions of leaf sizes and leaf morphologies indicate that as the leaf size increases with time, the leaf shape gradually becomes rounder (Fig. S1), contributing to an increase in the efficiency of trapping CO_2_ efflux from the soil surface.

**Figure 2:**
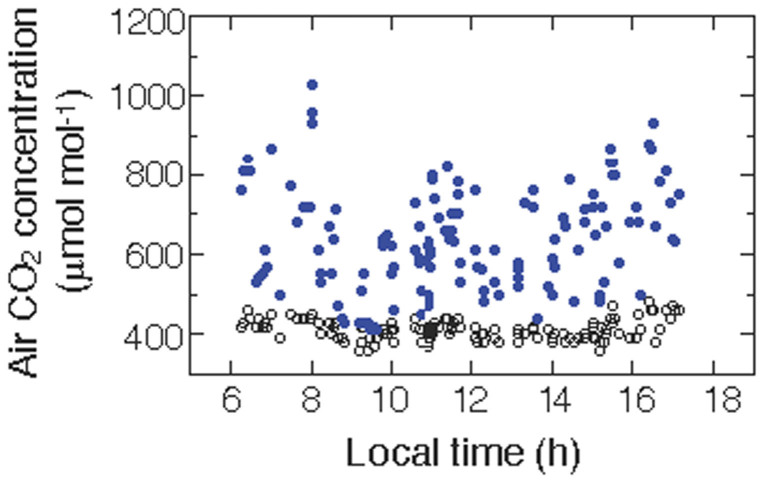
Diurnal time variations in air CO_2_ concentration at 20 cm above the ground (open circles) and in the air space between the leaf blade and soil surface (blue circles).

On a sunny day during the rainy season, the average daily [CO_2_] was 412 µmol mol^−1^ in the open air at 20 cm above the ground and 638 µmol mol^−1^ in the space between the leaves and soil surface (Fig. 2). The maximum [CO_2_] observed in the air space was greater than 1000 µmol mol^−1^. Nevertheless, [CO_2_] in the space largely fluctuated with temporal variations in wind velocity. The values (mean ± SD) of the stable carbon isotope ratios (δ^13^C) in the lamina were −34.9 ± 1.5 ‰ in the ginger plants and −29.1 ± 1.5 ‰ in the upper canopy leaves of woody plants in the dry evergreen forest (our unpublished data on woody plants). The low δ^13^C value in the ginger plants indicates high internal [CO_2_] in the leaves during the day.

**Figure 3:**
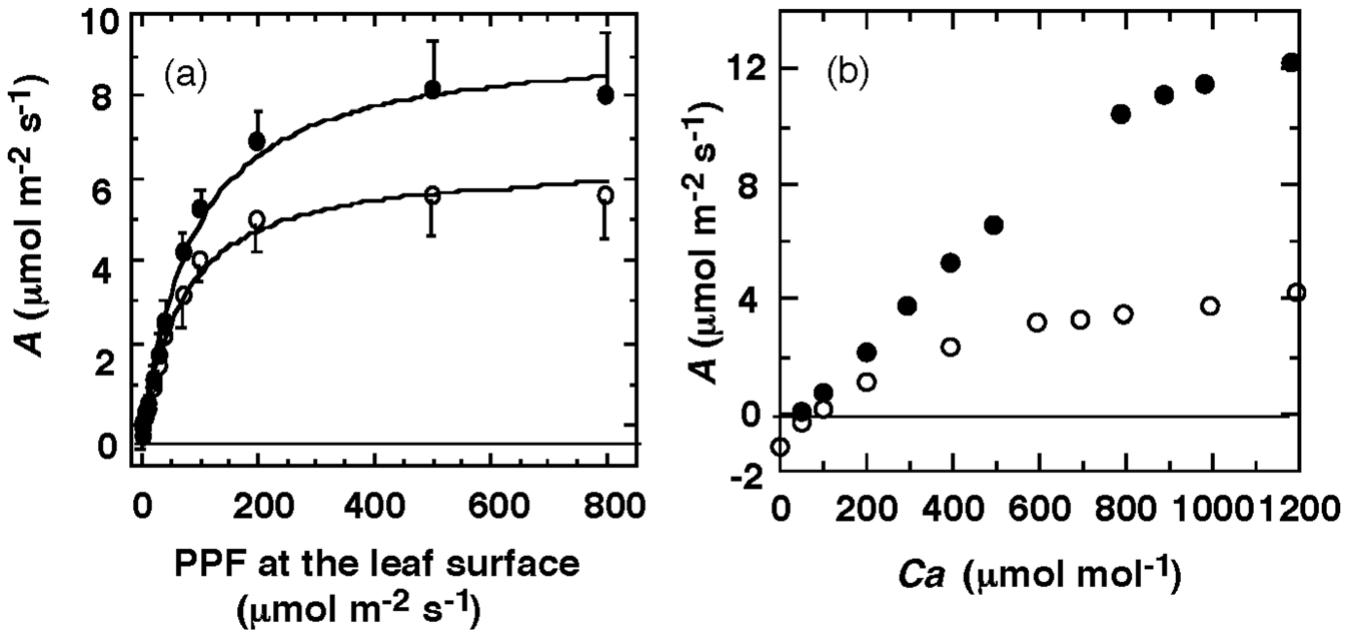
Photosynthetic responses under high CO_2_ concentrations and high light conditions. (a) Photosynthetic light response curves (PPF-*A* curves) at 600 µmol mol^−1^ CO_2_ (closed circles) and 400 µmol mol^−1^ CO_2_ (open circles), where PPF represents the photosynthetic photon flux at the leaf surface and *A* is net C assimilation rate. Bars indicate 1 SD, (b) Photosynthetic ambient-air CO_2_ response curves (*C*_a_-*A* curves) in a leaf blade at 500 µmol m^−2^ s^−1^ PPF (closed circles) and 40 µmol m^−2^ s^−1^ PPF (open circles), where *C*_a_ represents CO_2_ concentration in outlet gas stream in the LI-6400, i.e., ambient air CO_2_ concentration.

When the ambient-air [CO_2_] was artificially increased from 400 to 600 μmol mol^−1^, the *A* under near-light saturated conditions (800 µmol m^−2^ s^−1^ PPF: photosynthetic photon flux) increased from 5.8 to 8.2 μmol m^−2^ s^−1^, a 45% increase (Fig. 3A). In contrast, under low light conditions (less than 70 µmol m^−2^ s^−1^ PPF), no significant increase was detected in *A* after elevating [CO_2_] from 400 to 600 μmol mol^−1^. We also measured ambient-air CO_2_ response curves under 500 and 40 µmol m^−2^ s^−1^ PPFs. Both RuBP carboxylation and RuBP regeneration rates were reduced by the low PPF (Fig. 3B). When the ambient-air [CO_2_] was increased from 400 to 600 μmol mol^−1^, *A* increased by 48% under relatively strong sunlight (500 μmol m^−2^ s^−1^ PPF) and by 36% under reduced light (40 μmol m^−2^ s^−1^ PPF) conditions. The data indicate that a significant increase in *A* in response to elevated [CO_2_] was more pronounced under sunlit conditions compared with shaded conditions. Sunflecks must thus cooperate with rising [CO_2_] for enhancing of *A*^12^,^13^.

## Discussion

The data presented here indicate that the unique leaf structure of ginger plant enhances C fixation under high light conditions by effectively trapping high [CO_2_] efflux in the relatively closed space between their leaves and the soil surfaces. In tropical forests, high termite activity at ground level prevents fallen leaves from covering the leaf surface of the ginger plants (Fig. 1A); the leaf litter layer typically remains fairly thin and does not persist for a long period of time. This may be a factor in explaining why the ginger plant has evolved to capture CO_2_ efflux from soil respiration in tropical forests.

Another unique morphological characteristic of the ginger plant is the small root system (Fig. 1B). Large non-photosynthetic organs are found to have large respiration requirements^18^,^19^. However, its small root system, the ginger plant has a very low CO_2_ compensation point at the whole plant level, similar to leafy plants^14^. Because of the small root system, the ginger plant can only grow during the favorable rainy season as an ephemeral plant. Another advantage is the high soil respiration during rainy seasons. In tropical dry forests in Thailand, where the ginger plant is a native species, the soil respiration rates become double during the rainy seasons^20^. The mean soil respiration rate is approximately 7.67 µmol m^−2^ s^−1^ in the rainy season and approximately 3.63 µmol m^−2^ s^−1^ in the dry season.

A relatively high irradiance is required to effectively enhance *A* under elevated [CO_2_] (Fig. 3B); light levels greater than approximately 6.4% of full sunlight appear to be required to maintain a population of the ginger plant (see Environmental description in Supplementary information). Under sunlit conditions, the risk of photoinhibition increases even in tropical climates, particularly in shaded plants at relatively high temperatures^21^,^22^. However, in the ginger plant, xanthophyll-cycle dependent non-photochemical quenching (NPQ) appears to prevent chronic photoinhibition (Fig. S3). This unique adaptation to specific microhabitats is reflected by the plant distribution. In the tropical dry forests, the ginger plant is primarily located in the drought-deciduous forests with sparse tree cover and lightly shaded forest floors. In contrast, the ginger plant is exclusively located on the edges of dry evergreen forests with closed canopies.

The discovery of the morphological adaptation of the ginger plant is the first demonstration of the effective use of high CO_2_ efflux from soil in understory C_3_ plants. Their unique structure of this plant is characterized by large, flat leaves, thus earning the nickname “terrestrial water lily”. The shape delimits the space between the leaves and the ground surface (Fig. 1A). Plants with such an ideal leaf structure are rare even in the tropics. We suggest that the C_3_ ginger plant evolved to cope with low atmospheric CO_2_ by morphologically trapping high CO_2_ efflux from the soil, whereas C_4_ plants did so by physiologically concentrating CO_2_ within the plant body. In adult trees of certain woody plants, the respiration rates per unit stem surface at breast height ranges from 1.2 µmol m^−2^ s^−1^ to 3.5 µmol m^−2^ s^−1^^23^,^24^,^25^. The CO_2_ efflux from the stem surface is due to numerous parenchyma cells located within stems^18^, carbon transport in phloem from the leaves to the roots^26^, and CO_2_ up-flow from root systems due to transpiration-driven sap flow^20^,^27^,^28^,^29^. Although the stem respiration rates of large trees are reduced compared with than the soil respiration rates during the rainy season, stem respiration may be valuable as a CO_2_ source for living plants. Therefore, a mechanism similar to that of the ginger plant may be identified among the lichens, mosses, ferns, orchids, and vines growing not only on the ground but also on the trunks of large trees. We hypothesize that the combination of a closed air space and relatively high sunlight is required to exploit extremely high efflux CO_2_.

The pulse-labeling method has been used to determine the time lag from CO_2_ efflux from soil to leaf C assimilation^30^. The time lag ranges from 12.5 ± 7.5 (mean ± SD) h in grasses to 4 to 5 days in trees. Although the data indicate that interactions between the soil and plants in the C cycles within a single ecosystem exist, most CO_2_ that originates from the soil will have dissipated from the ecosystem by diffusion during this time period. The low δ^13^C values of ginger plants indicate that they were exposed to high [CO_2_] and used large amounts of C emitted from the soil. Nevertheless, shady conditions increase internal [CO_2_] in leaves due to the reduced *A*, consequently decreasing the δ^13^C values in laminae^31^. Therefore, we cannot use δ^13^C values to distinguish between the two potential sources of the effects, shade and high ambient air [CO_2_]. Overall, we can conclude that root and microbial-derived CO_2 _are major contributors to carbon assimilation in this ginger plant.

## Methods

The study was conducted in July 2008 in a dry evergreen forest in Thailand (14° 29′N, 101° 55′E, 563 m ASL) approximately 180 km northeast of Bangkok during the middle of the rainy season^32^. We selected a population of ginger plants found roadside in a forest with a dense canopy. During three successive days, the diurnal time courses of PPF, ambient air temperatures and relative humidity in air were measured near the center of the plant population (data shown in Fig. S2). On a relatively sunny day, the diurnal time courses of leaf gas exchange and chlorophyll fluorescence were measured from predawn to dusk using an open, portable measurement system (LI-6400, LI-COR, Lincoln, NE) and a chlorophyll fluorescence meter (Mini-PAM, Walz, Effeltrich, Germany), respectively. These measurements were conducted in eight individual plants with relatively large leaves.

While measuring diurnal leaf gas exchange, the diurnal variations in ambient air [CO_2_] were simultaneously measured with thin-film capacitance CO_2_ sensors (GM70, Vaisala, Helsinki, Finland) without tube-absorbing air. The CO_2_ sensors were set at two heights: 1) 20 cm above the ground and 2) in the air space between the leaf blade and the ground surface in an individual plant with a relatively large leaf area. The diameter of the CO_2_ sensor probe was 18.5 mm, and the leaf diameter was greater than 100 mm. Because of without tube-absorbing and given a large leaf, [CO_2_] in the air space below the leaf could be directly measured (Fig. S4); it is possible that we did not completely avoid air leaks along the side of the prove, possibly resulting in an underestimation of [CO_2_].

In the following days, to evaluate the interactive effects of light intensity and [CO_2_] on *A*, we measured photosynthetic light responses (PPF-*A* curve) under different ambient air [CO_2_] levels and photosynthetic ambient air CO_2_ responses (Ca-*A* curve) under different light levels during the daylight hours (Ca refers to ambient air [CO_2_]). To evaluate the average internal [CO_2_] in leaves over a long time period, carbon isotope ratios in the eight laminae were examined with an isotope ratio mass spectrometer (DELTA V Plus, Thermo Fisher Scientific Inc., Cambridge, UK). More detailed information is described in the supplementary information.

## Author Contributions

A.I. and T.N. designed and carried out the major part of the field measurements. M.A, K.Y., N.O. and P.L. carried out the field measurements. S.D. and L.P. designed and prepared the field works. A.I. and J.Y. wrote the manuscript.

## Supplementary Material

Supplementary Information Supplementary Information
