# Supplementary material for: Effective use of high CO2 efflux at the soil surface in a tropical understory plant
Source: Sci Rep. 2015 Mar 11;5:8991. doi: 10.1038/srep08991 (PMC4355870; doi:10.1038/srep08991)
Supplement: Supplementary Information [file srep08991-s1.pdf]

Supplementary Information

Title: Effective use of high CO_2 efflux at soil surface in a tropical understory plant

Authors: Atsushi Ishida, Takashi Nakano, Minaco Adachi, Kenichi Yoshimura, Noriyuki Osada, Phanumard Ladpala, Sapit Diloksumpun, Ladawan Puangchit, Jin Yoshimura

Corresponding Author: Atsushi Ishida (atto@ecology.kyoto-u.ac.jp)

Environmental description and more detailed methods

Study site and plant materials

The study was located in the dry evergreen forest at the Sakaerat Environmental Research Station (14° 29'N, 101° 55'E, 563 m ASL), approximately 180 km northeast of Bangkok in Thailand. Mean annual temperature was 26.2°C, and mean annual rainfall was 1240 mm (Sakurai *et al.* 1998). There is a distinct dry season from

November to March (Ishida *et al.* 2006). The soil is of sandstone origin and acidic (around 4.5 in pH), and has relatively poor nutrients with high porosity. *Hopea ferrea* Lanessan (Dipterocarpaceae) is the predominant tree with tall canopies (approximately 25-35 m high) in the evergreen forest. Details in landform and soil characteristics are shown in Pitman (1996) and Murata *et al.* (2009).

The examined ginger plant (*Kaempferia marginata* Carey, Zingiberaceae) is a drought-deciduous perennial herb. The plants are usually found near the roadside in the dry evergreen forests with dense canopies, and inside the drought deciduous forests with sparse canopies in the Sakaerat Environmental Research Station. Thus, the ginger plant seems to favor to a relatively light understory.

Measurements of leaf size distribution

We selected a population of the ginger plant found roadside in the dry evergreen forest. To examine the ontogenetic variations of leaf form, we measured the leaf area, leaf length and width, and the top heights of the leaf blades in 150 individual plants with contrasting plants size. The data in leaf shape and the top height are shown in

Supplementary Fig. 1 and Supplementary Table 1, respectively.

Microclimate measurements

Photosynthetic photon flux (PPF) was measured with quantum sensors (LI-190SB, LI-COR, Lincoln, NE, USA) at an open place and two understory places around the center of a ginger plant population for three consecutive, relatively sunny days from July 15 to July 17 in 2008 in the middle of the wet season. PPF measurement at the open site was conducted on the top of a 45-m high scaffolding tower constructed near the study site, which exceeded the uppermost canopy in the forest. Ambient air temperature (T_{air}) and relative humidity (RH) in the understory were simultaneously measured with thermistor and thin-film capacitance sensors, respectively (Model 36355, Hioki-Denki, Nagano, Japan). These sensors were connected with small data loggers (Model 3631 or 3635, Hioki-Denki), and the data were minutely stored. These measurements in the understory were conducted at 20 cm above the ground near the center of the ginger population.

On a sunny day (July 15 in 2008), the diurnal variations in ambient air CO_2

concentrations ([CO_2]) were directly measured with thin-film capacitance CO_2 sensors (GM70, Vaisala, Helsinki, Finland) without tube-absorbing air. The CO_2 sensors were set at two heights: (1) 20 cm above the ground and (2) in the air space between the leaf blade and the ground surface in a lamina. To measure CO_2 concentrations in the air space, we selected an individual plant with a relatively large leaf area to avoid air leak along the side of the censor probe (Supplementary Figure 4).

Environmental description

Supplementary Figure 2 shows the diurnal time variations in microclimate (PPF, air temperature, and relative humidity in air) in the examined population growing at a roadside in the dry evergreen forest and the diurnal variations in PPF at an open place. The daily total PPF at the study site relative to that of the open site was 6.4%. The understory light levels in tropical evergreen forest are approximately 1% of full sunlight or sometimes less than 1% (e.g., Ashton 1992). Because the ginger plants are not found in deeper-shaded sites in the evergreen forests, the light levels of approximately 6.4% relative to full sunlight appear to be required to maintain a population of the ginger

plant.

More detailed methods in the photosynthetic capacity measurements

The leaf photosynthetic capacity in eight individuals was measured with an open, portable measurement system (LI-6400, LI-COR, Lincoln, NE). The measurement was conducted in six healthy, mature leaves. The leaf chamber with 6 cm^2 was used and the red-blue RED lamp unit was utilized as a light source. Photosynthetic light-response curves were measured on the same eight leaf blades under 600 and 400 μmol mol^{-1} CO_2 in the inlet gas stream with LI-6400. The values of 400 and 600 μmol mol^{-1} CO_2 were approximate and corresponded to daily mean air CO_2 concentrations at the 20 cm above and just below the leaf blades, respectively. PPFs were decreased stepwise from 800, 500, 200, 100, 70, 40, 30, 20, 10, 7, 3, to 0 μmol m^{-2} s^{-1}. Light compensation points and apparent quantum use efficiencies were calculated from linear regressions under very low PPFs (from 0 to 10 μmol m^{-2} s^{-1}). The mean leaf temperature during these measurements was 29.2°C which approximately corresponds to the daytime leaf temperature. Photosynthetic ambient air CO_2-response curves were measured on the

same seven leaf blades under 500 and 40 μmol m^{-2} s^{-1} PPF with LI-6400. In the values that were exposed to sun-flecks and in those without sunflecks during the daytime, the values of 500 and 40 μmol m^{-2} s^{-1} PPF were used, respectively (see Supplementary Fig. 2). The [CO_2] in the inlet gas stream with LI-6400 increased stepwise from 0, 50, 100, 200, 300, 400, 500, 600, 700, 800, to 1000 μmol mol^{-1}.

More detailed methods in the diurnal variations of leaf gas exchange and chlorophyll fluorescence measurements

The diurnal time change in leaf gas exchange was measured with an open, portable measurement system (LI-6400, LI-COR, Lincoln, NE), from dawn to dusk on 15 July 2008. The measurement was conducted in eight healthy, mature leaves at approximately 30-minute intervals. The leaf chamber with 6 cm^2 was used and the top part of the chamber was sealed with a clear plastic plate to receive naturally incident PPF. The CO_2 concentration in the inlet gas stream within LI-6400 was adjusted at 600 μmol mol^{-1}, which approximately corresponded to the mean air [CO_2] just below the leaf blades (see Figure 2).

While measuring the leaf gas exchange, the diurnal time variations in chlorophyll *a* fluorescence were measured with a fluorescence meter (Mini-PAM, Walz, Effeltrich, Germany), according to Bilger *et al.* (1995). The fiber-optic cable was connected with the clear top-cover of the LI-6400 chamber, while the angle (60°) and the distance between the leaf surface and the fiber-optic cable were manually adjusted. Maximum fluorescence yield (F_m) and dark fluorescence yield (F_o) in photosystem II (PSII) were determined just before dawn. Just after the measurement of leaf gas exchange, we supplied a saturated-light pulse to the leaf surface. Maximum fluorescence (F_m') and steady-state fluorescence (F) in the light-adapted state of PSII were measured during the daytime. Chlorophyll fluorescence parameters were calculated, according to Genty *et al.* (1989). The potential maximum quantum yield of PSII ($F_v/F_m = (F_m-F_o)/F_m$) was calculated from the dark-time measurements made before dawn. For each daytime measurement, the effective quantum yield of PSII ($\Phi_{PSII} = (F_m'-F)/F_m'$) was calculated. Assuming that photosystem I and II absorb equal amounts of light and the leaf absorbance of lamina is 0.84, the electron transport rate through PSII (ETR) was calculated as, ETR = 0.5 Φ_{PSII} 0.84 PPF (at the leaf surface).

Non-photochemical quenching (NPQ = (F_m/F_m')-1) was also calculated. Data in chlorophyll fluorescence are showed in Supplementary Figure 3.

Measurements of the nitrogen and stable carbon isotope ratio in lamina and the number and size of stomata

After all measurements, we collected the leaves and then cut leaf discs with a borer. The leaf discs were oven dried (70°C, 72 hr) and weighed to determine leaf dry mass per unit leaf area (LMA). The total nitrogen (N) and carbon (C) contents within the leaf discs were measured with an N-C analyzer (Sumigraph NC-900, Sumitomo-Kagaku, Osaka).

To estimate the averaged internal CO_2 concentrations in leaves for a long time, the stable carbon isotope ratios ($\delta^{13}C$) in lamina were determined with an isotope ratio mass spectrometer (DELTA V Plus, Thermo Fisher Scientific Inc., Cambridge, UK). The $\delta^{13}C$ values were expressed in delta notation relative to a PD Belemnite standard: $\delta^{13}C = (R_{sample} – R_{standard} - 1)\ 1000$ (‰), where R_{sample} is the $^{13}C/^{12}C$ ratios of the samples and $R_{standard}$ is the $^{13}C/^{12}C$ ratio of the standard.

The numbers and the pore length of stomata in the adaxial and abaxial leaf surfaces were determined by obtaining replicas of the surface of four healthy leaves with a celluloid plate (Universal Micro-printing, SUMP, Tokyo, Japan).

References

Ashton, P. M. S. Some measurements of the microclimate within a Sri Lankan tropical rainforest. *Agri. Forest Meteoro*. **59**, 217-235 (1992).

Bilger, W., Schreiber, U. & Bock, M. Determination of the quantum efficiency of photosyntem II and of non-photochemical quenching of chlorophyll fluorescence in the field. *Oecologia* **102**, 425-432 (1995).

Genty, B., Briantais, J. -M. & Baker, N. R. The relationship between the quantum yield of photosynthetic electron transport and quenching of chlorophyll fluorescence. *Biochem. Biophys. Acta* **990**, 87-92 (1989).

Ishida, A., Diloksumpun, S., Ladpala, P., Staporn, D., Panuthai, S., Gamo, M., Yazaki, K., Ishizuka, M. & Puangchit, L. Contrasting seasonal leaf habits of canopy trees between tropical dry-deciduous and evergreen forests in Thailand. *Tree Physiol.* **26**,

643-656 (2006).

Murata, N., Ohta, S., Ishida, A., Kanzaki, M., Wachirinrat, C., Archawakom, T. & Sase, H. Comparison of soil depths between evergreen and deciduous forests as a determinant of their distribution, Northeast Thailand. *J. Forest Res.* **14**, 212-220 (2009).

Pitman, J. I. Ecophysiology of tropical dry evergreen forest, Thailand: measured and modelled stomatal conductance of *Hopea ferrea*, a dominant canopy emergent. *J. Appl. Ecol.* **33**, 1366-1378 (1996).

Sakurai, K., Tanaka, S., Ishizuka, S., Kanzaki, M. Differences in soil properties of dry evergreen and dry deciduous forests in the Sakaerat Environmental Research Station. *Tropics* **8**, 61-80 (1998).

Supplementary Table 1 Leaf morphological and physiological characteristics in *Kaempferia marginata* Carey.

Variables	unit	mean	1 SD
Top height of leaf blads above the ground	mm	24	13
Area of individual leaf blads	cm^2	65.0	36.9
Length/width ratio in individual leaf blads		1.32	0.30
Leaf dry mass per leaf area (LMA)	$g\ m^{-2}$	35.8	4.7
Nitrogen (N) content per leaf area	$mmol\ N\ m^{-2}$	54.8	6.4
N concentration per leaf dry mass	$mmol\ N\ g^{-1}$	1.54	0.15
C/N ratio in leaf blades		19.9	2.05
Stomatal pore length	μm	39.75	6.37
Stomatal density in adaxial leaf surface	$No.\ mm^{-2}$	1.6	0.6
Stomatal density in abaxial leaf surface	$No.\ mm^{-2}$	21.0	4.3
Light compensation point	$\mu mol\ m^{-2}\ s^{-1}$	2.5	1.5
Apparent quantum use efficiency		0.071	0.008
Area-based dark respiration rates	$\mu mol\ m^{-2}\ s^{-1}$	-0.17	0.08

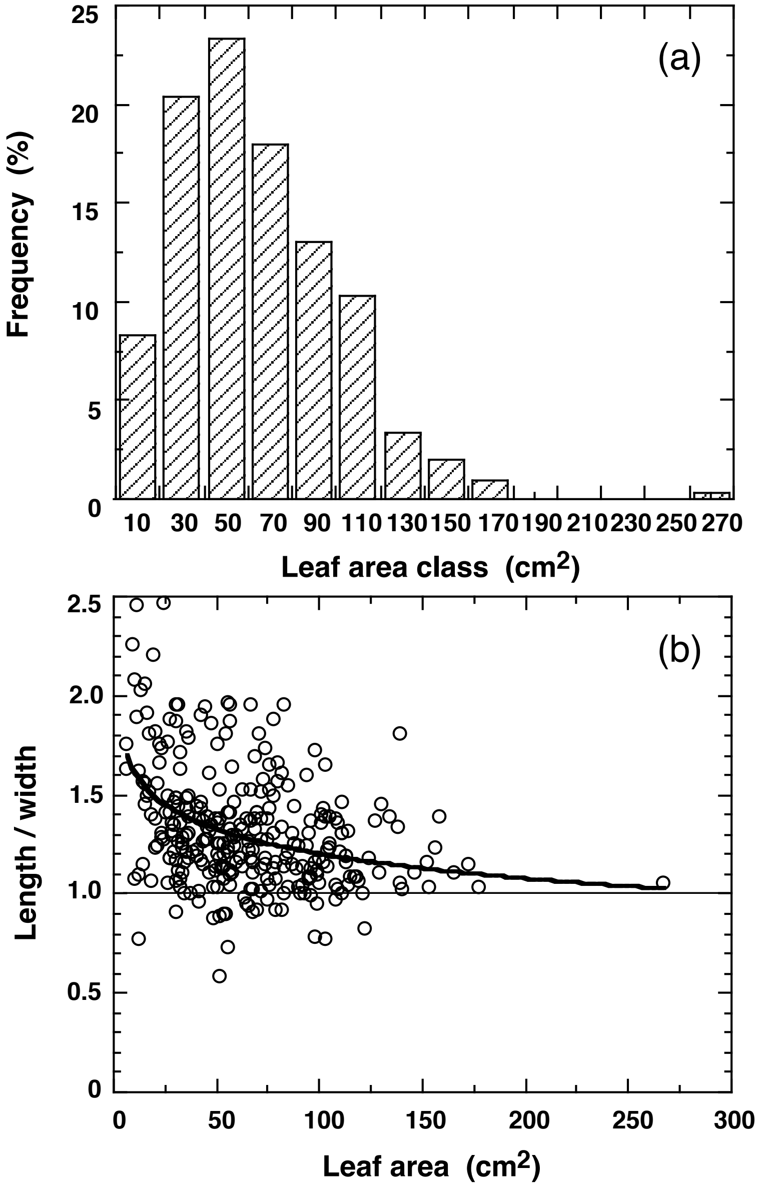

Supplementary Figure 1: The ontogenetic variations in size and shape of leaf blades in 150 ginger individuals with different plant sizes. (a) The frequency of blade size of single leaves, and (b) the change of leaf shape (the ratio of length to width in each leaf blade) with leaf area.

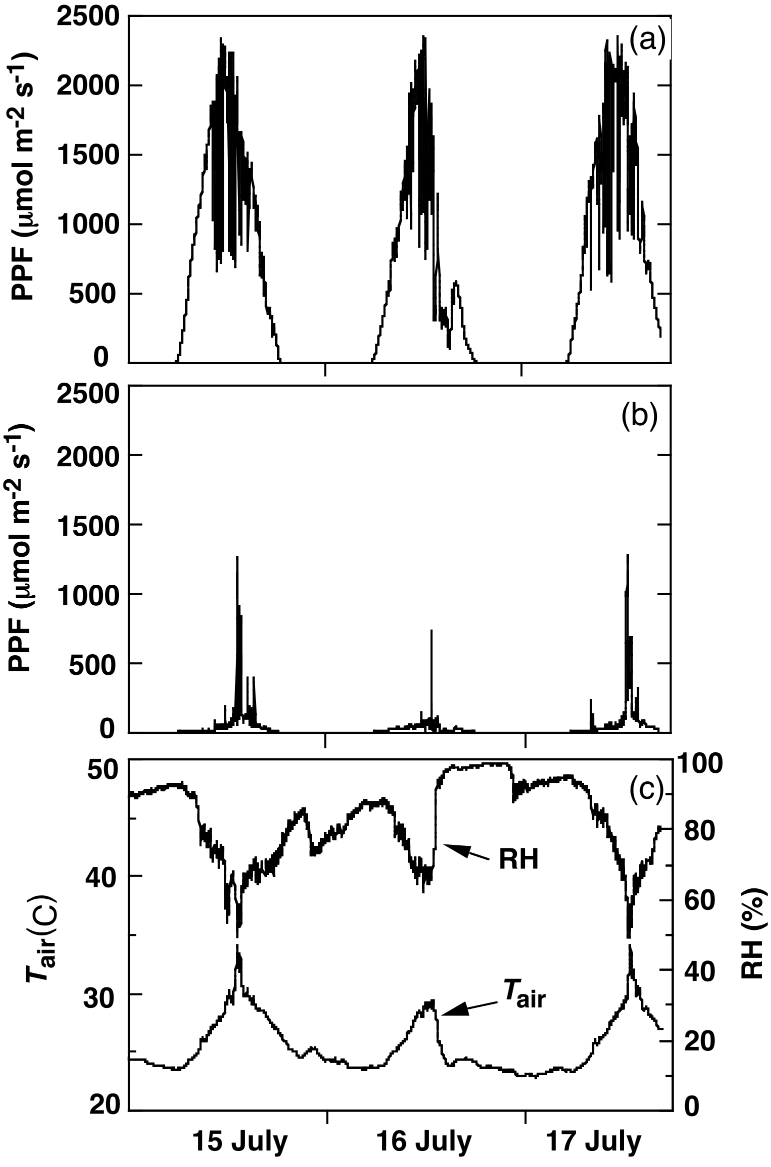

Supplementary Figure 2: The diurnal time courses in microclimate at the understory during the successive three days from on 15 July to 17 July (the mid-rainy season) in 2008. Incident photosynthetic photon flux (PPF) at (a) an open place and (b) the understory (20 cm above the ground), and (c) air temperature (T_{air}) and relative humidity (RH) in air at an understory site.

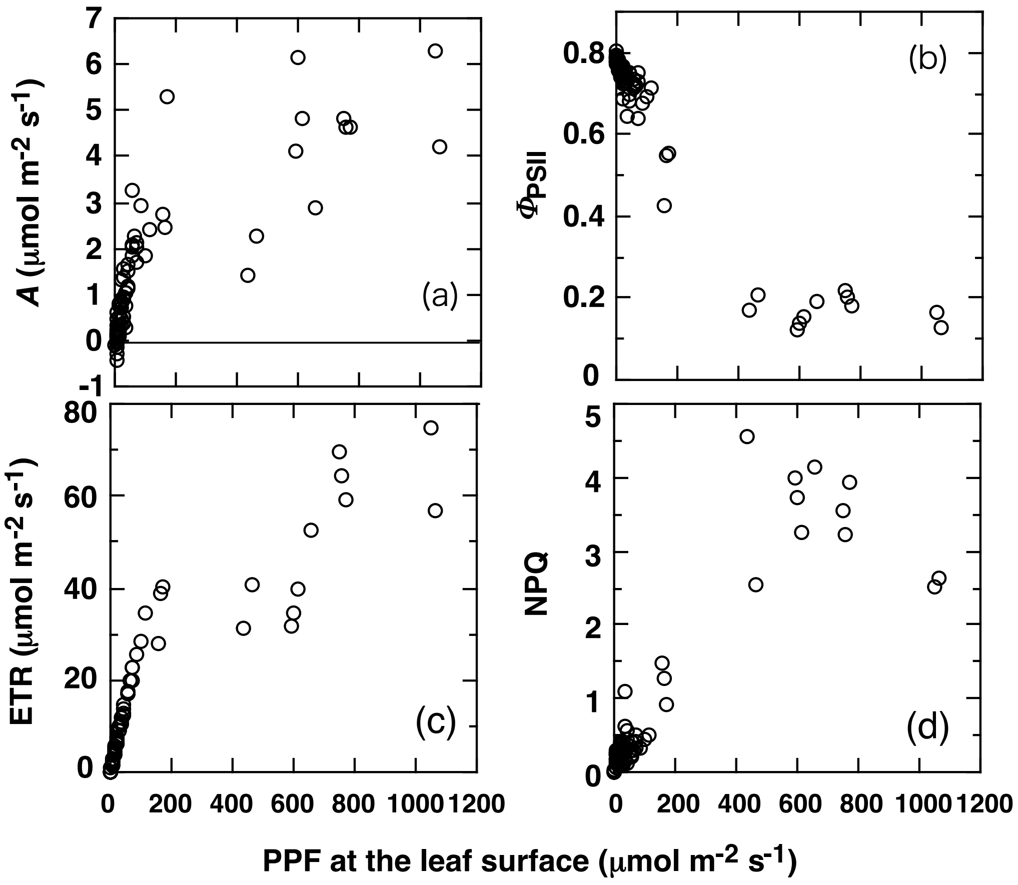

Supplementary Figure 3: The relationships between photosynthetic capacity and photosynthetic phone flux (PPF) at the leaf surface. Data were obtained from the measurements of diurnal time courses in (a) net photosynthetic rates, (b) PSII quantum yield, (c) electron transport rates through PSII, and (d) Stern-Volmer non-photochemical quenching coefficient.

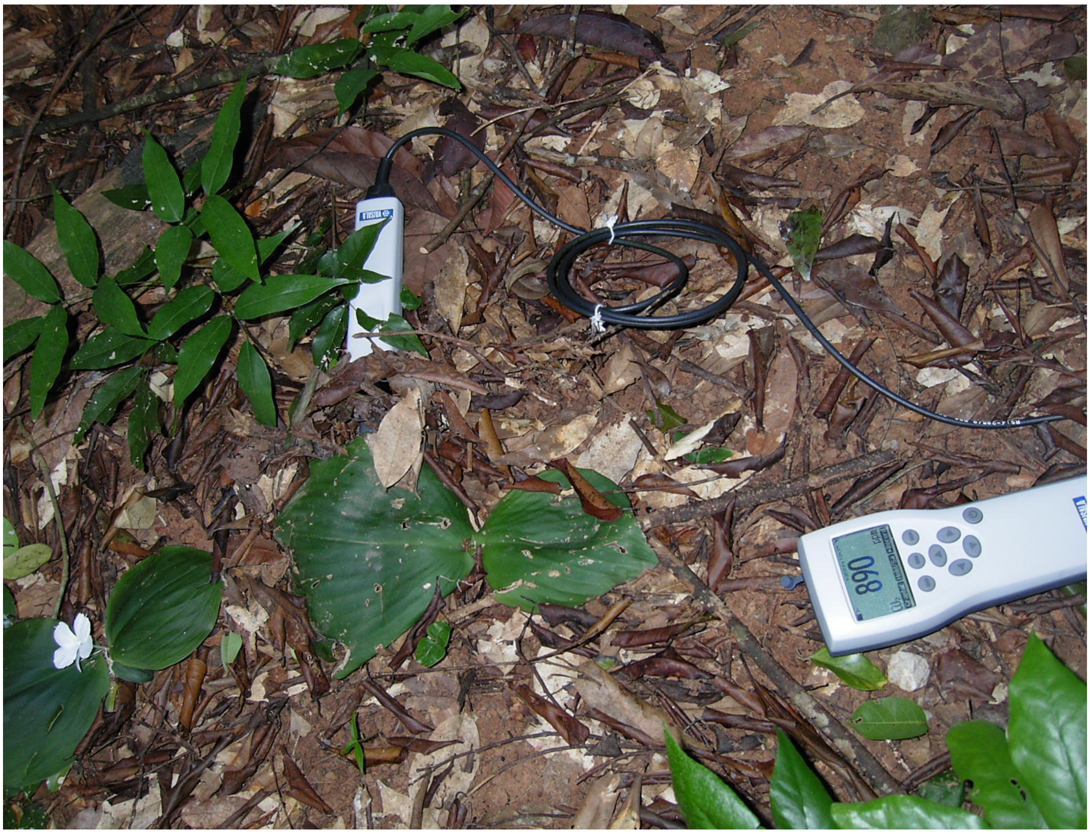

Supplementary Figure 4: The measurement of CO_2 concentrations in the air space between the leaf blade and the soil surface.
